# Respiratory Microbiota Profiles Associated With the Progression From Airway Inflammation to Remodeling in Mice With OVA-Induced Asthma

**DOI:** 10.3389/fmicb.2021.723152

**Published:** 2021-08-30

**Authors:** Jun Zheng, Qian Wu, Ya Zou, Meifen Wang, Li He, Sheng Guo

**Affiliations:** ^1^Department of Traditional Chinese Medicine, Shanghai Children’s Hospital, Shanghai Jiao Tong University, Shanghai, China; ^2^Department of Emergency Medicine, Putuo Hospital, Shanghai University of Traditional Medicine, Shanghai, China; ^3^Department of Pediatrics, Sanmen People’s Hospital, Taizhou, China; ^4^Department of Endocrine, Genetics and Metabolism, Shanghai Children’s Hospital, Shanghai Jiao Tong University, Shanghai, China

**Keywords:** asthma, respiratory microbiota, airway inflammation, airway remodeling, metagenomic functional prediction

## Abstract

**Background:**

The dysbiosis of respiratory microbiota plays an important role in asthma development. However, there is limited information on the changes in the respiratory microbiota and how these affect the host during the progression from acute allergic inflammation to airway remodeling in asthma.

**Objective:**

An ovalbumin (OVA)-induced mouse model of chronic asthma was established to explore the dynamic changes in the respiratory microbiota in the different stages of asthma and their association with chronic asthma progression.

**Methods:**

Hematoxylin and eosin (H&E), periodic acid-schiff (PAS), and Masson staining were performed to observe the pathological changes in the lung tissues of asthmatic mice. The respiratory microbiota was analyzed using 16S rRNA gene sequencing followed by taxonomical analysis. The cytokine levels in bronchoalveolar lavage fluid (BALF) specimens were measured. The matrix metallopeptidase 9 (MMP-9) and vascular endothelial growth factor (VEGF-A) expression levels in lung tissues were measured to detect airway remodeling in OVA-challenged mice.

**Results:**

Acute allergic inflammation was the major manifestation at weeks 1 and 2 after OVA atomization stimulation, whereas at week 6 after the stimulation, airway remodeling was the most prominent observation. In the acute inflammatory stage, *Pseudomonas* was more abundant, whereas *Staphylococcus* and *Cupriavidus* were more abundant at the airway remodeling stage. The microbial compositions of the upper and lower respiratory tracts were similar. However, the dominant respiratory microbiota in the acute inflammatory and airway remodeling phases were different. Metagenomic functional prediction showed that the pathways significantly upregulated in the acute inflammatory phase and airway remodeling phase were different. The cytokine levels in BALF and the expression patterns of proteins associated with airway remodeling in the lung tissue were consistent with the metagenomic function results.

**Conclusion:**

The dynamic changes in respiratory microbiota are closely associated with the progression of chronic asthma. Metagenomic functional prediction indicated the changes associated with acute allergic inflammation and airway remodeling.

## Introduction

Bronchial asthma is one of the most common chronic respiratory diseases globally. At present, it affects more than 300 million individuals worldwide, and its incidence continues to increase (Huang et al., 2019). According to estimates, the global medical cost of asthma is as high as US $100 billion per year (Rothenberg, 2016). While asthma affects all age groups, it is frequently diagnosed in children. The major symptoms of asthma include coughing, wheezing, shortness of breath, and chest tightness, which are associated with variable expiratory airflow impairment due to bronchoconstriction, increased mucus production, and airway remodeling.

Asthma occurrence and development are related to several factors, including genetic predisposition, immunity, and environmental factors, among others. Although the pathogenesis of asthma is yet to be fully understood, allergic airway inflammation is considered one of the leading causes of allergic asthma (Xiao et al., 2021). Lymphocytes, particularly helper T lymphocytes, are known to play an important role in airway inflammation in asthma (Tumes et al., 2017). To date, most studies have shown that an imbalance in the Th1/Th2 and CD4 + CD25 + Treg/Th17 cell counts is closely related with the occurrence and development of asthma. An increase in the levels of Th2 cytokines (IL-4, IL-5, and IL-13, among others) and Th17 cytokines (IL-17 and IL-6, among others) was observed in both patients with asthma and animal models of asthma (Gandhi et al., 2020). Furthermore, Treg cytokines (IL-10 and TGF-β, among others) were reduced in patients with asthma and animal models of asthma (Boonpiyathad et al., 2019). As the inflammatory response persists in asthma, it eventually leads to airway remodeling. The primary characteristics of airway remodeling are subepithelial collagen deposition, hyperplasia and hypertrophy of airway smooth muscles, angiogenesis under the mucosa, and hyperplasia of mucus glands, which eventually lead to the thickening of the airway wall and narrowing of the functional cavity (Fehrenbach et al., 2017).

In recent years, with the development of high-throughput sequencing technology, the relationship between changes in the human microbiota and asthma has attracted increasing attention. The “hygiene hypothesis” was the first theory to suggest a link between microbes and allergy (Strachan, 1989). Th2 cell-mediated airway inflammation in ovalbumin (OVA)-stimulated germ-free mice was considerably stronger than that in mice colonized with symbiotic microorganisms. The alterations in the immune response driven by microbes indicate that microbial exposure affects the risk of asthma. Dysbiosis and the subsequent dysregulation of microbiota-related immunological processes affect the onset of the disease, its clinical characteristics, and treatment responses (Barcik et al., 2020). For a long time, the lower respiratory tract (LRT) was considered to be sterile; hence, studies on the link between microbes and asthma have mostly focused on the gut microbiota. However, in recent years, several studies have shown that the respiratory microbiome is also closely associated with the occurrence and development of asthma (Budden et al., 2019). The predominant respiratory microbial phyla are Proteobacteria, Firmicutes, Bacteroidetes, and Actinobacteria. Proteobacteria, the most common respiratory microbes in patients with asthma, can promote neutrophil aggregation and are closely associated with airway hyperresponsiveness (AHR) and airway inflammation (Huang et al., 2011, 2015). Previous studies have shown that at the genus level, the abundance of *Haemophilus*, *Neisseria*, and *Fusobacterium* increased significantly in the respiratory tract of patients with asthma (Durack et al., 2017). A 16S rRNA gene sequencing study on the nasal microbiota of children aged less than 5 years showed that the increased abundance of *Streptococcus*, *Haemophilus*, and *Moraxella* was an important risk factor for predicting wheezing in preschoolers that persisted till a school-going age (Teo et al., 2018). *In vitro*, the epithelial damage and inflammatory cytokine expression (IL-33 and IL-8) induced by *Moraxella catarrhalis* were considerably greater than that induced by dominant nasal bacterial isolates (McCauley et al., 2019). *Bifidobacterium* polysaccharides can inhibit Th17-related inflammation in the lungs (Schiavi et al., 2016). *Haemophilus influenzae* is the most common pathogen detected in patients with asthma (Wood et al., 2010). The P38 MAPK pathway was found to be activated when macrophages isolated from bronchoalveolar lavage fluid (BALF) specimens collected from patients with asthma were cultured with *Haemophilus parainfluenza in vitro* (Goleva et al., 2013). In addition, some lung microbes also produce metabolites (such as short-chain fatty acids) and play a specific biological role (Remot et al., 2017; Segal et al., 2017).

However, only a limited number of studies have investigated the association between the dynamic changes in respiratory microbiota and the chronic progression of asthma or whether the changes in respiratory microbiota may trigger asthma progression. In this study, an OVA-induced mouse model of chronic asthma was established to investigate the differences in respiratory microbiota at different stages of asthma development and their association with the chronic progression of asthma using metagenomic functional prediction; we believe the findings will help identify new strategies for the prevention and treatment of asthma in the future.

## Materials and Methods

### Reagents

Ovalbumin was purchased from Sigma Chemical Co. (OVA; grade V; St. Louis, MO, United States). Imject Alum was obtained from Thermo Fisher Scientific (Rockford, IL, United States). Mouse IL-4, IL-6, IL-10, and IL-17A ELISA kits were purchased from R&D Systems (Minneapolis, MN, United States). Mouse total IgE and OVA-specific IgE ELISA kits were purchased from BioLegend (San Diego, CA, United States). Antibodies against matrix metallopeptidase 9 (MMP-9) and vascular endothelial growth factor (VEGF-A) were obtained from Abcam Co. (Cambridge, MA, United States).

### Animal Experiments

Female BALB/c mice (n = 30) aged 4–6 weeks and weighing 17–20 g were obtained from Shanghai Sippr-BK Laboratory Animal Co. Ltd. (Shanghai, China). All mice were maintained in a specific-pathogen-free environment under controlled conditions with a 12 h light/dark cycle at an appropriate temperature and humidity. All animal experiments were conducted in accordance with the institutional guidelines for animal research. The experimental protocol was approved by the Center for Laboratory Animals, Shanghai Children’s Hospital, Shanghai Jiao Tong University, Shanghai, China (approval no. 2017Y003).

**FIGURE 1:**
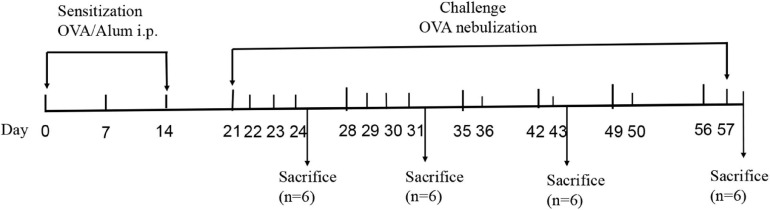
Sensitization-challenge protocols for mice with OVA-induced asthma.

The mouse model of OVA-induced chronic asthma was established as described previously with minor modifications (Temelkovski et al., 1998). Briefly, the 30 mice were divided into two groups: the control group and OVA group. The mice in the OVA group were sensitized by intraperitoneally injecting with 200 μL of a solution composed of 20 μg OVA dissolved in 100 μL sterile saline and 100 μL Imject Alum [containing 4 mg aluminum hydroxide (net weight)] on days 0, 7, and 14. For the OVA challenge, the mice were exposed to an OVA aerosol [2.5% (wt/vol) OVA solution in sterile saline administered using a PARI PRONEB Ultra compressor (Pari Proneb, Midlothian, WA, United States)] for 30 min on days 21–24 and 28–31, followed by exposure two times per week for an additional 4 weeks. Mice in the control group (*n* = 6) were injected intraperitoneally with saline and an Imject Alum emulsion and then exposed to an aerosol of sterile saline without OVA according to the same schedule. Six mice in the OVA group were sacrificed in the first (OVA1W), second (OVA2W), fourth (OVA4W), and sixth weeks (OVA6W), and the mice in the control group were sacrificed at the end of the experiment. The mice were sacrificed within 24 h of the final nebulization. After the mice were euthanized, BALF, nasal lavage fluid (NLF), and the left lower lung tissues were collected and preserved. The sensitization-challenge protocols used in this study are summarized in Figure 1.

### Histological Analysis and Immunofluorescence Staining of Lung Tissue

We first ligated the left lower lung and removed it after complete bronchoalveolar lavage washing. The lungs were harvested, infused with 4% paraformaldehyde, and maintained for 24 h. Sections (4 μm-thick) were embedded in paraffin and then subjected to hematoxylin and eosin (H&E), periodic acid-schiff (PAS), and Masson’s trichrome staining to evaluate airway inflammation, mucus production, and collagen deposition in the lung tissues. Pathological changes were observed using a light microscope at 200× magnification. To grade the extent of lung inflammation, goblet cell hyperplasia and collagen fiber deposition semiquatitative scoring system was used as previously described (Kujur et al., 2015; Kubo et al., 2019). Pathological scoring was performed by three different individuals blinded to the experimental methods.

Immunofluorescent staining was performed to measure MMP-9 and VEGF-A expression in the lung tissue specimens. The paraffin-embedded sections were cut and mounted. The sections were deparaffinized and rehydrated using a standard protocol. Next, the tissue sections were treated with a citric acid antigenic repair solution for antigenic repair and washed with PBS three times after cooling. The sections were permeabilized by treatment with 0.5% Triton X-100 for 30 min at room temperature. Then, the sections were incubated with 3% BSA for 30 min at room temperature, followed by immunostaining with primary antibodies (anti-MMP-9 antibody, 1:250; anti-VEGF-A antibody, 1:200) overnight at 4°C. IgG isotype controls were used for each group of tissue samples at the same concentration. The slides were washed with PBS and labeled with fluorescent tagged goat anti-rabbit secondary antibody (1:1,000) for 1 h in the dark at room temperature. The slides were then treated with DAPI (1:200), a fluorescent nuclear stain for improved protein localization. The slides were washed three times with PBS, rinsed with PBS, mounted, sealed, and evaluated within 2 h of staining under an epifluorescence microscope. Representative fluorescent images of each slide were acquired at 200× for morphometric and comparative analysis.

### Collection of BALF and NLF

After the mice were sedated with low doses of 10% chloral hydrate, and euthanized by cervical dislocation, their lungs were washed four times by endotracheal intubation to the LRT with cold sterile saline (0.5 mL in each round) to collect BALF specimens. Then, a sterile leather hose was re-inserted to rinse the nasal cavity with normal saline (0.5 mL in each round) for 3–4 times and to collect the NLF. The BALF and NLF were filtered once using a 0.22 μm filter. The filtered BALF was centrifuged at 1,000 × *g* for 15 min, and the supernatants were collected for cytokine detection. The membrane of the filter was used for subsequent DNA extraction. All specimens were temporarily stored at –80°C.

### Cytokine and IgE Measurement

The BALF specimens were collected as described above. The levels of IL-4, IL-6, IL-10, IL-17A, total IgE, and OVA-specific IgE in BALF were measured using ELISA kits according to the manufacturer’s instructions.

### 16S rRNA Gene Sequencing and Bioinformatics Analysis

To explore the association between the dynamic changes in respiratory microbiota and the progression of chronic asthma, 16S rRNA gene sequencing was performed using NLF and BALF specimens from control, OVA1W, OVA2W, and OVA6W asthmatic mice.

Total genomic DNA was extracted using the OMEGA Soil DNA Kit (Omega Bio-Tek, Norcross, GA, United States) according to the manufacturer’s instructions and stored at –80°C before further analysis. PCR amplification of the V3–V4 region of bacterial 16S rRNA genes was performed using the forward primer 338F (5′-ACTCCTACGGGAGGCAGCA-3′) and the reverse primer 806R (5′-GGACTACHVGGGTWTCTAAT-3′). Sample-specific barcodes were incorporated into the primers for multiplex sequencing. The PCR mixture contained 5 μL of reaction buffer (5×), 0.25 μL of Fast Pfu DNA Polymerase (5 U/μL), 2 μL (2.5 mM) of dNTPs, 1 μL (10 μM) each of the forward and reverse primers, 1 μL of the DNA template, and 14.75 μL of ddH_2_O. The thermal cycling conditions were as follows: initial denaturation for 2 min at 98°C, 25 cycles of denaturation for 15 s at 98°C, annealing for 30 s at 55°C, extension for 30 s at 72°C, and a final extension step of 5 min at 72°C. The PCR amplicons were purified using Vazyme VAHTSTM DNA Clean Beads (Vazyme, Nanjing, China) and measured using the Quant-iT PicoGreen dsDNA Assay Kit (Invitrogen, Carlsbad, CA, United States). After the individual quantification step, the amplicons were pooled in equal quantities, and paired-end sequencing (2 × 300 bp) was performed using Illumina MiSeq (Illumina, San Diego, CA, United States) at Shanghai Personal Biotechnology Co., Ltd. (Shanghai, China).

Microbiome bioinformatics analysis was performed using QIIME 2 2020.11 (Hall and Beiko, 2018) with minor modifications (recommended in the official tutorials). Briefly, raw sequence data were demultiplexed using the demux plugin, followed by primer cutting with the cutadapt plugin. The sequences were subjected to quality filtering, denoising, and merging, and the chimera were removed using the DADA2 plugin (Callahan et al., 2016). Non-singleton amplicon sequence variants were aligned using MAFFT and used to construct a phylogeny with FastTree (Price et al., 2009). We used the q2-diversity plugin for computing different α diversity metrics using Shannon’s diversity index and β diversity metrics using weighted UniFrac distance matrices. Principal coordinates analysis (PCoA) with weighted UniFrac distance matrices was used to study the community composition. Taxonomy was assigned using a Naive Bayes classifier pre-trained on the Greengenes 13_8_99% OTUs 16S rRNA gene full-length sequences and the q2-feature-classifier plugin (Bokulich et al., 2018). Linear discriminant analysis effect size (LEfSe; Segata et al., 2011) was used to detect differentially abundant taxa across groups using the default parameters. The PICRUSt software package (Langille et al., 2013) was used for metagenomic functional prediction analysis. Kyoto Encyclopedia of Genes and Genomes (KEGG) pathways (Kanehisa et al., 2004) were used to identify the metagenomic contents. LEfSe and PICRUSt analysis were performed using the resource available at http://huttenhower.sph.harvard.edu/galaxy/. The STAMP software (Parks et al., 2014) was used to analyze the predicted metagenome and identify pathways associated with asthma.

### Statistical Analysis

The expression levels of IgE, inflammatory factors, and airway remodeling-related proteins were compared by analysis of variance using SPSS 24.0 and GraphPad Prism 8.0. In case of statistical significance, the two groups were compared using a least significant difference *t*-test. The Kruskal–Wallis test was used to estimate intergroup differences for α diversity metrics, β diversity metrics, and LEfSe analysis. The Wilcoxon test was used for comparison between subclasses. The predicted metagenome was analyzed using White’s non-parametric *t*-test for comparison between two groups. Statistical tests used in the study were two-sided, and a *P* value ≤0.05 was considered to indicate statistical significance.

## Results

### While the Increase in Airway Inflammation Is Not Obvious, Airway Remodeling Increases Gradually in Mice With OVA-Induced Chronic Asthma

**FIGURE 2:**
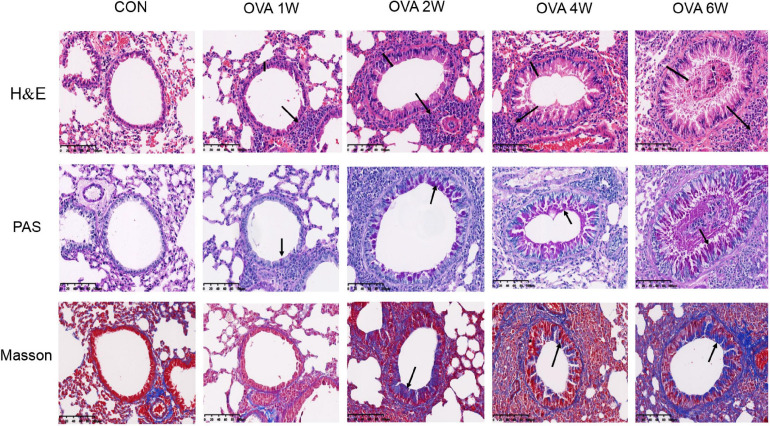
Changes in the pulmonary pathology of mice with OVA-induced chronic asthma. Representative hematoxylin and eosin (H&E)-stained lung sections showing inflammatory cell infiltration around the small airways, bronchial wall thickening, and constriction (200×; the black arrows indicate the aggregation of inflammatory cells, and the black vertical line indicates the airway wall thickness). PAS staining indicating the mucus-producing goblet cells around the small airways (200×; similar to the purple area indicated by the black arrow). Masson’s trichome staining indicating collagen fiber deposition around small airways (200×; similar to the blue area indicated by the black arrow).

As indicated by the H&E, compared to the control mice, OVA-challenged mice showed obvious inflammatory cell infiltration around the small airways, bronchial wall thickening, and constricted, even occluded, lumen. As the time period of the OVA challenge increased, there were no significant increases in the signs of allergic inflammation (Figure 2 and Supplementary Figure 1A). PAS staining showed that the number of mucus-secreting goblet cells increased gradually from OVA2W to OVA6W mice (Figure 2 and Supplementary Figure 1B). We observed that collagen fiber deposition around the small airways increased gradually and significantly in the OVA-challenged mice (starting with OVA2W mice) compared to that in control mice, as indicated by Masson’s trichome staining (Figure 2 and Supplementary Figure 1C). This indicated the increase in the degree of pulmonary fibrosis and the gradual progression of airway remodeling.

### Diversity of the Respiratory Microbiota in Mice With OVA-Induced Chronic Asthma

**FIGURE 3:**
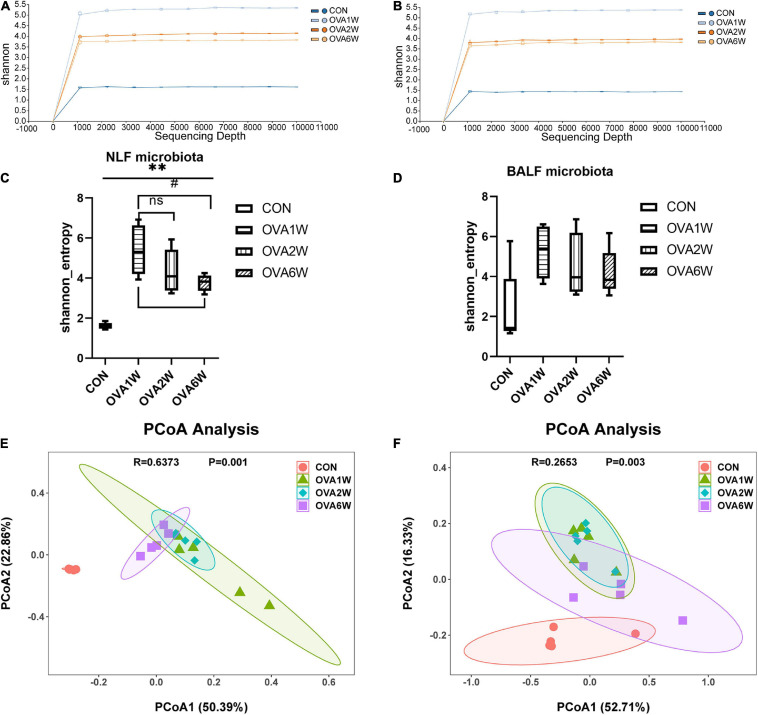
Diversity of the respiratory microbiota in mice with OVA-induced chronic asthma. **(A)** The alpha rarefaction curve in NLF microbiota; **(B)** The alpha rarefaction curve in BALF microbiota; **(C)** α diversity analysis (using Shannon index) of the NLF microbiota; **(D)** α diversity analysis (using Shannon index) of the BALF microbiota; **(E)** PCoA plot showing the β diversity of NLF microbiota (*P* = 0.001); **(F)** PCoA plot showing the β diversity of BALF microbiota (*P* = 0.003). PCoA of all samples using weighted UniFrac distance. PCoA, principal coordinates analysis. (*n* = 5 in each group).

The shape of the alpha rarefaction curve indicated that the sequencing depth in this study was sufficient (Figures 3A,B). The α diversity indicates the richness, diversity, and evenness of the species in a locally homogeneous habitat. The upper respiratory tract (URT) microbiota (NLF samples) of the OVA-induced mice had a significantly higher α diversity (*P* < 0.05) than that of the control mice, as determined using the Shannon index (Figure 3C). However, there were no significant differences between the Shannon indices of LRT microbiota (BALF samples) from the control and OVA-induced asthmatic mice (*P* > 0.05) (Figure 3D).

The β diversity among the different groups was evaluated using the weighted UniFrac distance. The scatter plot based on the PCoA scores showed the clear separation of the community composition between the control and OVA-induced groups. PCoA revealed significant differences in the diversity of the respiratory microbiota of the OVA-induced and control mice (Figures 3E,F).

### Modifications in the Composition of the Respiratory Microbiota in Mice With OVA-Induced Chronic Asthma

**FIGURE 4:**
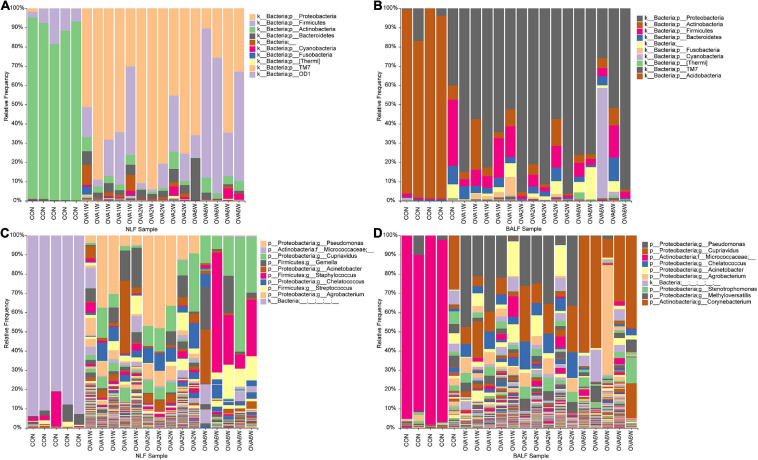
Microbial composition in the URT and LRT at the phylum and genus levels. **(A)** The composition of URT microbiota (NLF samples) at the phylum level. **(B)** The composition of LRT microbiota (BALF samples) at the phylum level. **(C)** The composition of URT microbiota (NLF samples) at the genus level. **(D)** The composition of the LRT microbiota (BALF samples) at the genus level (*n* = 5 in each group; only the top 10 legends with high abundance are shown).

To understand the differences between the respiratory microbiota in normal mice and OVA-induced asthmatic mice, we analyzed the microbial composition at the phylum and genus levels. We found that Proteobacteria, Firmicutes, Actinobacteria, and Bacteroidetes formed the four dominant phyla in all groups. At the phylum level, Actinobacteria were predominant in normal mice, whereas Proteobacteria, Firmicutes, and Bacteroidetes were the top three phyla in asthmatic mice (in both URT and LRT samples). There were no significant differences in the three dominant phyla in asthmatic mice at different time periods (Figures 4A,B).

At the genus level, the top three genera in the respiratory microbiota were *Micrococcaceae*, *Cupriavidus*, and *Pseudomonas*. *Micrococcaceae* had the highest abundance in control mice, whereas *Cupriavidus* and *Pseudomonas* were more abundant in asthmatic mice (Figures 4C,D).

**FIGURE 5:**
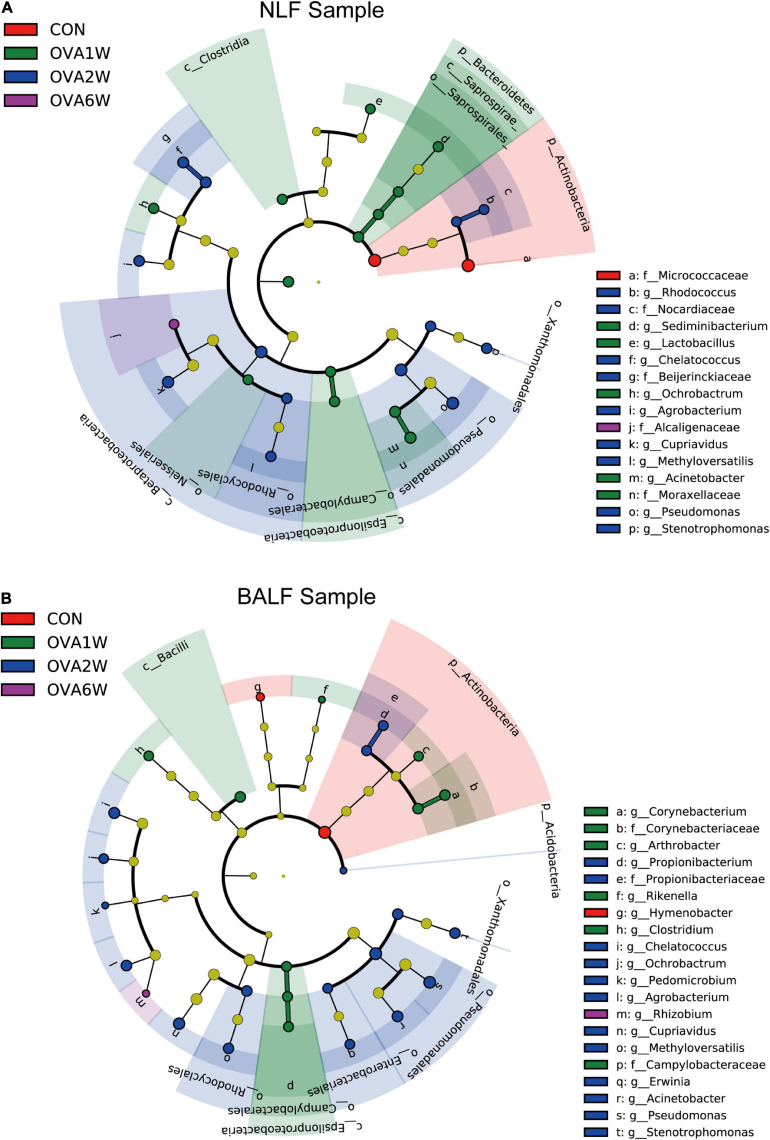
Different abundances of bacterial communities in the respiratory samples, as indicated in LEfSe analysis. The differences are indicated by the color of over-represented taxa: red indicating control mice, green indicating OVA1W mice, blue indicating OVA2W mice and purple indicating OVA6W mice. **(A)** Different abundances of bacterial communities in the URT (NLF samples) with LDA scores > 2.6. **(B)** Different abundances of bacterial communities in the LRT (BALF samples) with LDA scores > 2.5. The circles represent phylogenetic levels from phylum (innermost circle) to genera (outermost circle). *n* = 5 in each group; adjusted *P* values ≤ 0.05.

Interestingly, the predominant respiratory tract microbiota in the OVA-induced mice varied at different time points. To identify the differences in the bacterial community composition in different groups, we analyzed the different abundances of bacterial communities using LEfSe (Figure 5). The analysis of microbial composition at the genus level using LEfSe revealed that among URT microbiota, *Micrococcaceae* from Actinobacteria were most abundant in the control group, *Pseudomonas* from Proteobacteria in the OVA1W and OVA2W groups, and both *Cupriavidus* from Proteobacteria and *Staphylococcus* from Firmicutes in the OVA6W group. Among LRT microbiota, *Micrococcaceae* were most abundant in the control group, *Pseudomonas* in the OVA1W and OVA2W groups, and *Cupriavidus* in the OVA6W group. The results showed that the composition of the URT and LRT microbiota was similar; however, the composition of respiratory microbiota differed significantly at different stages of asthma.

### Metagenomic Functional Prediction of the Respiratory Microbiota of Mice With OVA-Induced Chronic Asthma

To understand whether the dynamic changes in the respiratory microbiota contribute to the progression of OVA-induced chronic asthma, we performed bacterial metagenomic function prediction analyses using the PICRUSt program. We explored the differences in bacterial metagenomic functional prediction among different groups at KEGG Level 3.

**FIGURE 6:**
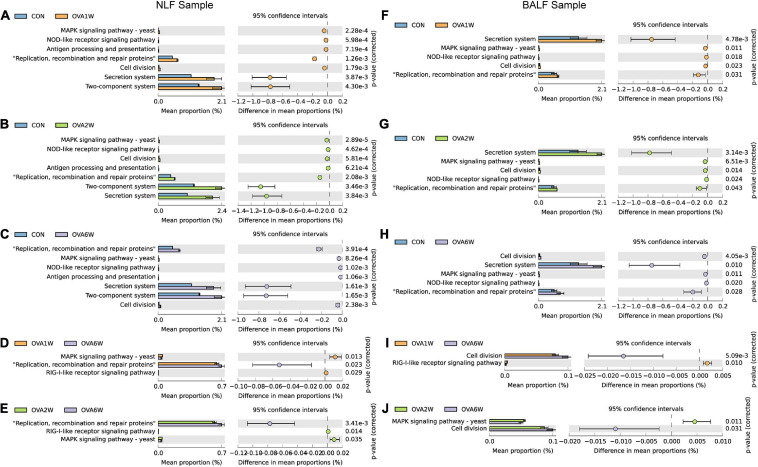
Metagenomic functional prediction of the respiratory microbiota using PICRUSt. Bacterial metagenomic functional categories were derived from level 3 KEGG pathways. Gene functions with a significant difference (corrected *P* value < 0.05) and parts of the pathways associated with asthma are shown. **(A)** Control group vs OVA1W group in URT samples. **(B)** Control group vs OVA2W group in URT samples. **(C)** Control group vs OVA6W group in URT samples. **(D)** OVA1W group vs OVA6W group in URT samples. **(E)** OVA2W group vs OVA6W group in URT samples. **(F)** Control group vs OVA1W group in LRT samples. **(G)** Control group vs OVA2W group in LRT samples. **(H)** Control group vs OVA6W group in LRT samples. **(I)** OVA1W group vs OVA6W group in LRT samples. **(J)** OVA2W group vs OVA6W group in LRT samples.

The following trends were observed in the URT samples. The pathways “MAPK signaling pathway-yeast” and “NOD-like receptor signaling pathway” (both associated with immune inflammation), “antigen processing and presentation,” and “two-component system” (associated with allergy) were significantly upregulated in the OVA1W and OVA2W group than in the control group (Figures 6A,B). Compared to the control group, “secretion system,” “replication, recombination and repair proteins,” and “cell division” were upregulated in the OVA6W group (Figure 6C). Comparison of the OVA1W and OVA2W groups with the OVA6W group (Figures 6D,E) showed that the immune inflammation-related pathways, such as “MAPK signaling pathway-yeast” and “RIG-I-like receptor signaling pathway,” were significantly upregulated in the OVA1W and OVA2W groups, whereas “replication, recombination and repair proteins” was upregulated in the OVA6W group.

The results predicted for the URT and LRT samples from the OVA and control groups at different time points were similar (Figures 6F–H). In the LRT samples, relative to the OVA6W group, “RIG-I-like receptor signaling pathway” was upregulated in the OVA1W group and “MAPK signaling pathway-yeast” was upregulated in the OVA2W group, whereas the pathway “cell division,” which is involved in recombination and repair, was upregulated in the OVA6W group (Figures 6I, J).

### Levels of IgE and Inflammatory Cytokines in BALF of Mice With OVA-Induced Chronic Asthma

**FIGURE 7:**
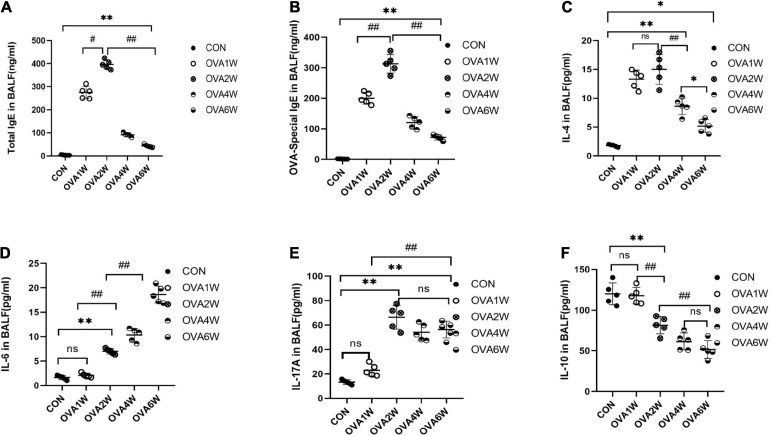
Levels of IgE and inflammatory cytokines in BALF samples. BALF was collected from control mice and OVA-challenged asthmatic mice to measure the levels of total IgE **(A)**, OVA-specific IgE **(B)**, IL-4 **(C)**, IL-6 **(D)**, IL-17A **(E)**, and IL-10 **(F)** using ELISA. Data are expressed as mean ± SD, tested using one-way ANOVA. *n* = 5, *P ≤ 0.05 vs the control group, **P ≤ 0.01 vs the control group; ^#^*P* ≤ 0.05 vs group OVA2W, ^##^*P* ≤ 0.01 vs group OVA2W. ns: no difference.

Metagenomic functional prediction revealed that the pathways associated with immune inflammation were significantly upregulated in the OVA1W and OVA2W groups; the total IgE, OVA-specific IgE, IL-4, IL-17A, IL-6, and IL-10 levels in BALF were measured using ELISA to confirm the results (Figure 7). Compared to the control group, the OVA group had significantly high levels of total IgE (Figure 7A) and OVA-specific IgE (Figure 7B) in BALF (*P* < 0.01), with the highest level recorded in the OVA2W group. After the OVA challenge, the trends observed in the changes in IL-4 (Figure 7C) and IgE levels were similar; however, there was no significant difference between the values obtained in the OVA1W and OVA2W groups. Among OVA-challenged mice, the IL-6 (Figure 7D) levels increased gradually from that in OVA2W mice and peaked in OVA6W mice (*P* < 0.01); however, there was no difference between the OVA1W and control groups. With the exception of the OVA1W group, the other OVA groups showed a significant increase in IL-17A (Figure 7E) levels compared to the control group (*P* < 0.01), with the highest value obtained in the OVA2W group; however, there was no significant difference among the OVA2W, OVA4W, and OVA6W groups. The IL-10 (Figure 7F) level was significantly reduced in OVA-challenged mice compared to that in the control group (*P* < 0.01); the OVA1W mice were an exception.

### Expression of MMP-9 and VEGF-A Proteins in Lung Tissues of Mice With OVA-Induced Chronic Asthma

**FIGURE 8:**
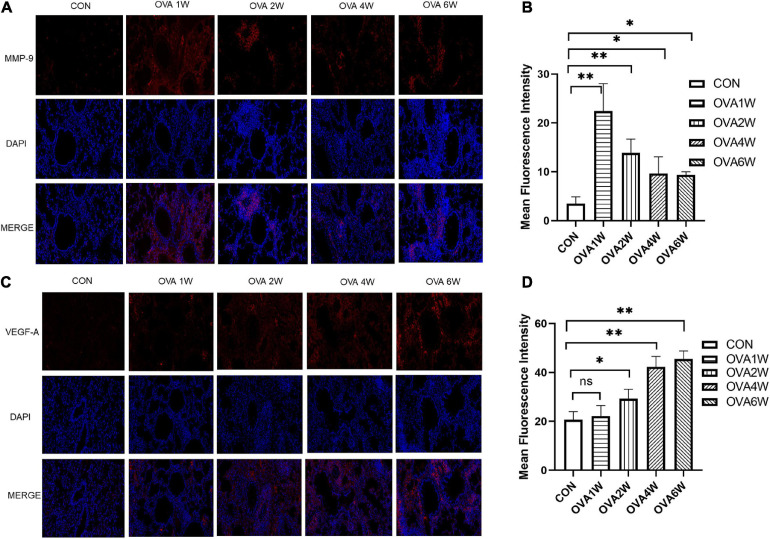
Expression of MMP-9 and VEGF-A proteins in lung tissues. **(A)** Immunofluorescent staining of MMP-9 (200×, red: MMP-9; blue: DAPI). **(B)** Mean fluorescence intensity of MMP-9. Each bar represents the mean ± SD (*n* = 6, *P ≤ 0.05 vs the control group; **P ≤ 0.01 vs the control group). **(C)** Immunofluorescent staining of VEGF-A (200×, red: VEGF-A; blue: DAPI). **(D)** Mean fluorescence intensity of VEGF-A. Each bar represents the mean ± SD (*n* = 6, *P ≤ 0.05 vs the control group; **P ≤ 0.01 vs the control group; ns: no difference).

Metagenomic functional prediction showed that the “replication, recombination and repair proteins” pathway was more frequently upregulated in the OVA6W group, and the expression levels of MMP-9 and VEGF-A in the lung tissues were measured using immunofluorescence to confirm this result. MMP-9 and VEGF-A are among the various mediators activated in airway remodeling. Compared to wild-type mice, MMP-9-deficient mice exhibited low levels of peribronchial fibrosis and lower total lung collagen content (Lim et al., 2006). VEGF-A transgenic mice exhibited an asthma-like phenotype with inflammation, vascular remodeling, mucus metaplasia, and AHR (Lee et al., 2004). Compared to the control mice, mice with OVA-induced asthma showed a significantly higher expression of MMP-9 protein around the small airways (*P* < 0.05). However, the expression of MMP-9 protein decreased gradually in these mice (Figures 8A,B). Compared to that in control mice, the expression of VEGF-A protein increased significantly in the lung tissues of OVA-challenged mice (*P* < 0.05), and the expression increased with time (Figures 8C,D).

## Discussion

In the present study, we explored the dynamic changes in the respiratory microbiota and their association with the progression of chronic asthma in a mouse model of OVA-induced chronic asthma. In previous studies on the respiratory microbiome, many differences were reported between patients with asthma and healthy people and between patients with different clinical types of asthma (Huang et al., 2011, 2015; Fazlollahi et al., 2018). However, there are no reports on the dynamic changes in the respiratory microbiota in patients with asthma or in animal models during disease progression. We first observed the dynamic changes in the lung pathology of the OVA-induced mice. At the early stage of asthma, the OVA-induced mice exhibited signs of inflammatory cell aggregation around the small airways. From the second week of nebulizing stimulation, small airway wall thickening and stenosis were increased gradually in the OVA-induced mice, following which the number of mucus-secreting goblet cells and collagen fibers increased; however, airway inflammation did not increase significantly. Airway remodeling was the major observation during the sixth week after atomization, whereas airway inflammation was the primary pathological change observed at the early stage of atomization stimulation in mice with OVA-induced chronic asthma. Since the lung pathology changes dynamically in OVA-induced mice, does the composition of the respiratory microbiota also change accordingly? We performed 16S rRNA gene sequencing to study the dynamic changes in the URT and LRT microbiota for the first time.

The microbial diversity analysis revealed that the α diversity, which represents the diversity of microbial groups in the model, increased drastically in the URT, whereas it remained unchanged in the LRT. However, the findings of most studies on the α diversity of respiratory microbiota are inconsistent (Goleva et al., 2013; Budden et al., 2019). β diversity analysis indicated the effect of OVA on the microbial diversities in different groups of mice. The results revealed the significant differences between the respiratory microbiota of OVA-induced mice at the acute airway inflammation and airway remodeling stages and that of control mice.

The β diversity indicated the differences in the respiratory microbiota of OVA-induced mice at different time points and that of normal mice; based on this finding, we studied the microbiota composition. The URT and LRT microbiota were similar at the phylum level, with Proteobacteria being the most abundant phylum and having a significantly higher abundance in OVA-induced mice than in normal mice at the acute inflammation and airway remodeling stages. Previous studies have shown that Proteobacteria have the highest abundance in both URT and LRT microbiota in patients with asthma (Huang et al., 2015; Li et al., 2017). Although the URT and LRT microbiota were shown to be highly consistent, Wypych et al. (2019) reported that URT samples do not accurately reflect the interactions that occur in the LRT, as the microbes present in the LRT depend on the balance between microbes that enter through the URT and those eliminated by host immunity. However, in our study, URT microbiota composition indicates the LRT microbiota composition at the phylum level to some extent. Proteobacteria were the predominant phylum in both URT and LRT specimens of OVA-induced asthmatic mice.

Since the phylum-level microbiota of OVA-induced mice was similar at different time periods, we analyzed the respiratory microbiota composition at the genus level. Among the URT microbiota, *Pseudomonas* was most abundant at the acute inflammatory stage, and *Staphylococcus* and *Cupriavidus* were most abundant at the airway remodeling stage. *Pseudomonas* was shown to be associated with the occurrence of asthma in several studies (Jung et al., 2016; Dimakou et al., 2018). Ferri et al. (2020) showed that the presence of *Pseudomonas* in sputum is an important risk factor for the persistence and frequent exacerbation of asthma. The abundance of *Staphylococcus* in the URT was shown to be significantly high in patients with asthma (Durack et al., 2018). Boutin et al. (2017) showed that the abundance of *Staphylococcus* was significantly higher in patients with pulmonary cystic fibrosis than in patients with asthma. This suggested that *Staphylococcus* may be more closely related to pulmonary fibrosis. *Cupriavidus* acts as an opportunistic pathogen in soil, and its association with asthma has been shown in previous studies. Zhao et al. (2020) showed that the abundance of *Cupriavidus* increased significantly in the URT of patients with asthma that were exposed to high concentrations of PM2.5 particulates. In the acute inflammatory phase, the bacteria with high abundance in the LRT and URT were similar, and only *Cupriavidus* was more abundant at the airway remodeling stage in the LRT. In general, we observed a high degree of similarity in the URT and LRT microbiota of OVA-induced mice. *Pseudomonas* was abundant at the acute inflammatory stage, whereas *Staphylococcus* and *Cupriavidus* were abundant at the airway remodeling stage. These results indicate that the predominant microbiota of the respiratory tract was different at different stages of asthma. *Pseudomonas* may play an important role in airway allergic inflammation, while *Staphylococcus* and *Cupriavidus* are closely related to the progress of airway remodeling.

To determine how the differences in microbiota composition influence the occurrence and development of airway inflammation and remodeling, we performed metagenomic function prediction. In both URT and LRT samples, the pathways associated with immune inflammation, including “MAPK signaling pathway-yeast,” “NOD-like receptor signaling pathway,” and “RIG-I-like receptor signaling pathway” were significantly upregulated during the acute inflammatory phase. The “antigen processing and presentation” and “two-component system” pathways associated with allergies were also upregulated at this stage. MAPK signaling was shown to be involved in airway inflammation and hyper-responsiveness in asthma (Bao et al., 2017; Sun X. et al., 2020; Sun Y. et al., 2020). The members of the NOD-like receptor family promote inflammatory cell recruitment and regulate immune responses in different tissues, such as lung tissues (Donovan et al., 2020). RIG-I was first shown to be associated with the innate immune response, and RIG-I-like receptor-induced signaling was shown to promote NF-κB and MAPK activation (Heine, 2011; Quicke et al., 2017; Lefkopoulos et al., 2020). The two-component system is a common and important signal transmission system in bacteria and is also a regulatory system for gene expression. It targets genes that encode proteins with bacteria-specific functions, including the hrp gene, which is an allergic response gene (Li et al., 2014; Piqué et al., 2015). At the airway remodeling stage, “secretion system” and “replication, recombination and repair proteins” were primarily upregulated, along with pathways related to recombination and repair, such as “cell division.” Previous metagenomic function prediction studies have only suggested significant differences in cellular processes, metabolism, genetic information processing, and human disease pathways between patients with asthma and healthy individuals (Millares et al., 2017). However, the metagenomic function prediction analysis of respiratory microflora at KEGG level 3 in animal models of asthma has not been reported. At a more in-depth level, our study confirmed the differences in metagenomic function prediction due to the differences in respiratory microbiota composition.

To confirm the predictions for respiratory microbiota, we measured the levels of total IgE, OVA-specific IgE, IL-4, IL-17A, IL-6, and IL-10 in BALF and the levels of airway remodeling-associated proteins in the lung tissues. The levels of total IgE, OVA-specific IgE, and IL-4 in BALF which represent levels of eosinophilic inflammation increased significantly during acute allergic inflammation. The levels of IL-6 and IL-17A in BALF and VEGF-A in lung tissues increased gradually. Previous studies have shown that IL-6 and IL-17A are associated with airway remodeling in asthma (Camargo et al., 2020; Hudey et al., 2020). The level of MMP-9 also increased significantly compared to that in normal mice, and the minor reduction in the MMP-9 level observed in the late stage could be attributed to the increased MMP-9 utilization during airway remodeling. The expression levels of these cytokines and proteins at different stages of asthma development were consistent with the metagenomic function prediction of respiratory microbiota.

This study had certain limitations. First, our findings are based on a small sample size study. Therefore, validity of the results will have to be confirmed in a larger sample size. Second, sample collection from the control mice and OVA-induced mice was not conducted at the same time points, and we were unable to eliminate the influence of age-related changes in the respiratory microbiota of the mice. Furthermore, the study duration was considerably short. Additionally, the atomization stimulation process was conducted in adult mice, and the mice had not entered senescence; hence, the bacterial and immune status in the mice did not change considerably. Thevaranjan et al. (2017) showed that senescent mice exhibited increased inflammation and changes in microbiota composition.

In conclusion, we established a mouse model of asthma from the onset of acute inflammation to the induction of airway remodeling, and observed the dynamic changes in respiratory microbiota for the first time. The dominant microbiota in the acute inflammatory and airway remodeling phases were different. The pathways associated with immune inflammation were significantly upregulated during the acute inflammatory phase. In contrast, different pathways, including “replication, recombination and repair proteins,” were upregulated at the airway remodeling stage. The cytokine levels in BALF and the concentration of airway remodeling-associated proteins in the lung tissues were consistent with the bacterial metagenomic function prediction. Although the precise relationship between airway inflammation and airway remodeling in patients with asthma is is not clear, these are important pathological mechanisms in the development of asthma (Banno et al., 2020). Our findings showed that in the progression of chronic asthma, the dominant microbiota at different stages are different, which may affect the pathophysiological processes in the host. These findings could form the basis for novel methods of asthma prevention and treatment.

## Data Availability Statement

The raw sequences of 20 female mice have been submitted to NCBI Project under accession number PRJNA730096 with NCBI Sequence Read Archive under accession number SRP320140.

## Ethics Statement

The animal study was reviewed and approved by Shanghai Children’s Hospital, Shanghai Jiao Tong University, Shanghai, China.

## Author Contributions

LH and SG conceived and designed the study, critically revised the manuscript, and were responsible for funding. JZ, QW, YZ, and MW completed animal experiments, acquired and interpreted the data, and drafted and critically revised the manuscript. All authors read and approved the final manuscript.

## Conflict of Interest

The authors declare that the research was conducted in the absence of any commercial or financial relationships that could be construed as a potential conflict of interest.

## Publisher’s Note

All claims expressed in this article are solely those of the authors and do not necessarily represent those of their affiliated organizations, or those of the publisher, the editors and the reviewers. Any product that may be evaluated in this article, or claim that may be made by its manufacturer, is not guaranteed or endorsed by the publisher.

## Supplementary Material

Supplementary Figure 1 The pathological score in the mice with OVA-induced chronic asthma. **(A)** Inflammation scores are based on H&E staining; **(B)** The PAS scores indicating the mucus-producing goblet cells around the small airways; **(C)** Masson staining score indicating collagen fiber deposition around small airways. Data are expressed as mean ± SD. *n*=5, ^∗^*P* ≤ 0.05 vs the control group, ^∗∗^*P* ≤ 0.01 vs the control group; ^#^*P* ≤ 0.05 between the two groups, ^##^*P* ≤ 0.01 between the two groups, ns: no difference.

Supplementary Figure 2 The leukocyte cell type counts in BALF samples. The BALF was centrifuged and the precipitated cells were counted after staining by Wright-Giemsa. Data are expressed as mean ± SD. *n*=5, ^∗^*P* ≤ 0.05 vs the control group, ^∗∗^*P* ≤ 0.01 vs the control group; ^#^*P* ≤ 0.05 between the two groups, ^##^*P* ≤ 0.01 between the two groups, ns: no difference.
