# Supplementary figures and images for: Respiratory Microbiota Profiles Associated With the Progression From Airway Inflammation to Remodeling in Mice With OVA-Induced Asthma
Source: Front Microbiol. 2021 Aug 30;12:723152. doi: 10.3389/fmicb.2021.723152 (PMC8435892; doi:10.3389/fmicb.2021.723152)

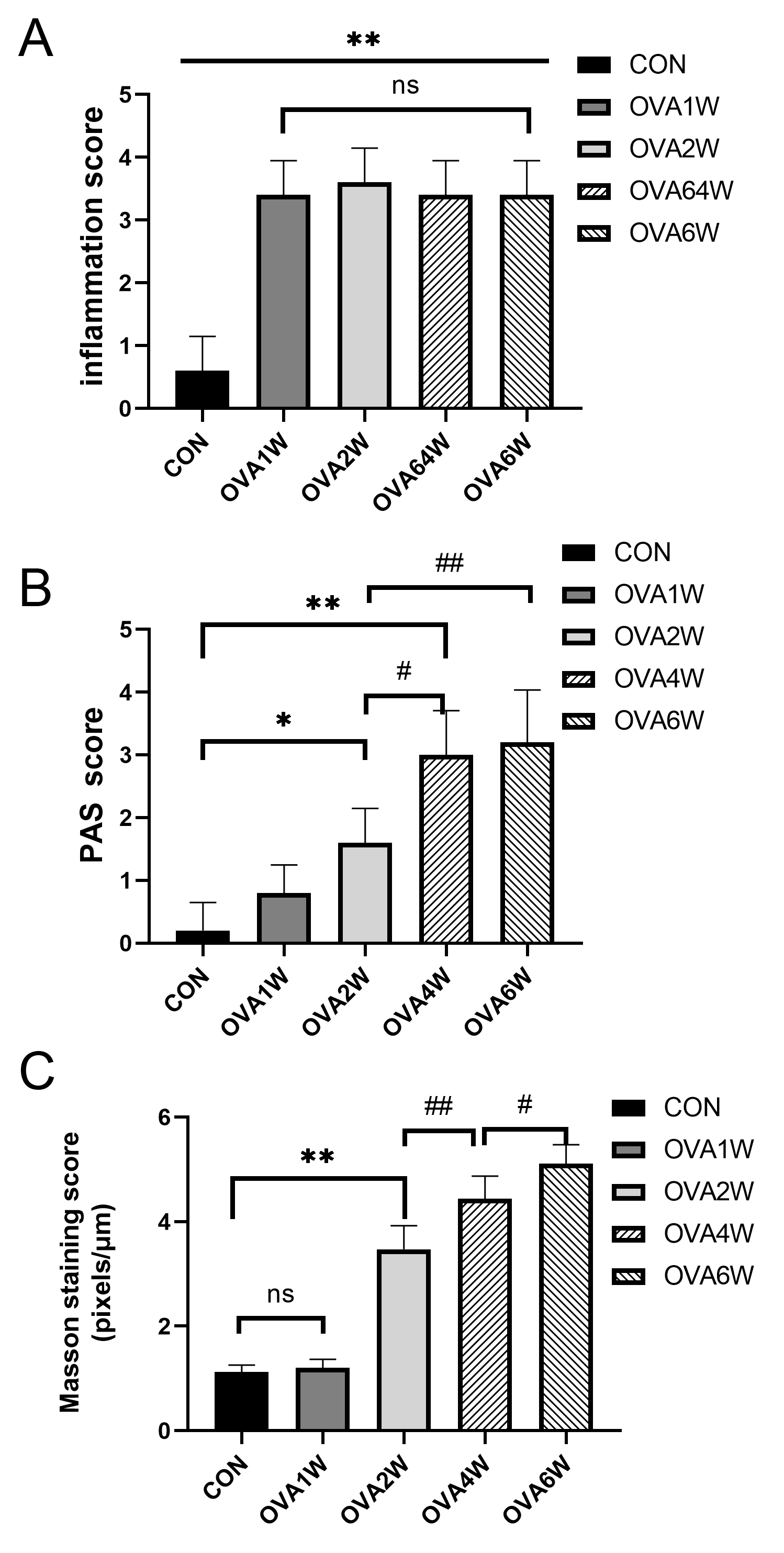

Supplement: Supplementary Figure 1 — The pathological score in the mice with OVA-induced chronic asthma. (A) Inflammation scores are based on H&E staining; (B) The PAS scores indicating the mucus-producing goblet cells around the small airways; (C) Masson staining score indicating collagen fiber deposition around small airways. Data are expressed as mean ± SD. n=5, ∗P ≤ 0.05 vs the control group, ∗∗P ≤ 0.01 vs the control group; #P ≤ 0.05 between the two groups, ##P ≤ 0.01 between the two groups, ns: no difference. [file Image_1.tif]

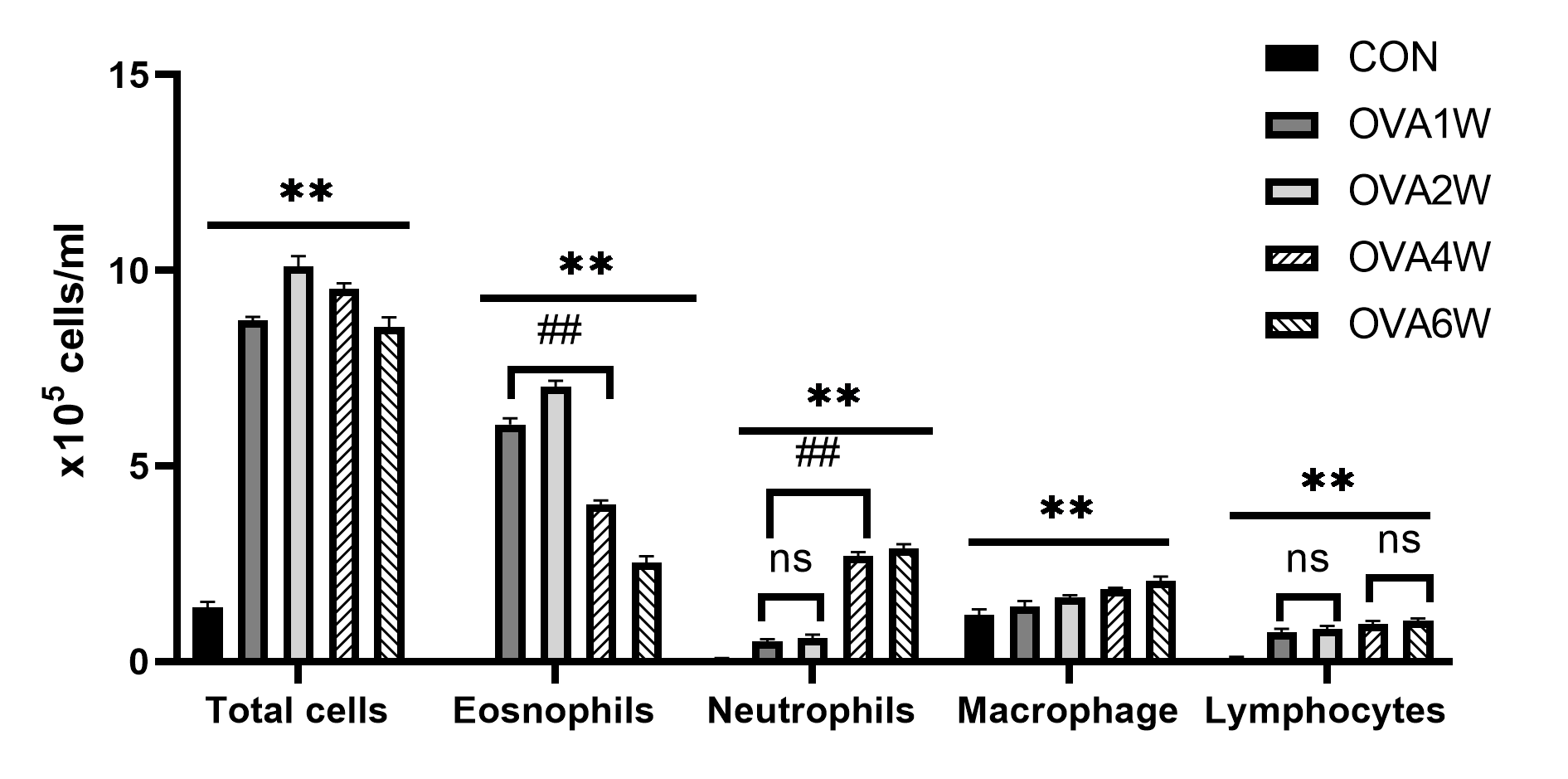

Supplement: Supplementary Figure 2 — The leukocyte cell type counts in BALF samples. The BALF was centrifuged and the precipitated cells were counted after staining by Wright-Giemsa. Data are expressed as mean ± SD. n=5, ∗P ≤ 0.05 vs the control group, ∗∗P ≤ 0.01 vs the control group; #P ≤ 0.05 between the two groups, ##P ≤ 0.01 between the two groups, ns: no difference. [file Image_2.tif]
